# Relationship between mandibular third molars and mandibular angle and condylar fractures

**DOI:** 10.4317/medoral.26604

**Published:** 2024-05-25

**Authors:** Jingying Mu, Yuna Wu, Chunfeng Wu, Huxiong Piao, Bin Jin

**Affiliations:** 1 Department of Stomatology, Baicheng Medical College, Jilin Baicheng, China; 2 Department of Stomatology, Yanbian University Hospital, Jilin Yanji, China; 3 Department of Post-Doctoral Research Center, Yanbian University Hospital, Yanji, Jilin Province, China; 4 Department of Cardiology, Yanbian University Hospital, Yanji, Jilin Province, China; 5 The 32183 Troops Hospital of PLA, China

## Abstract

**Background:**

Assess the correlation between the position of the third molar (M3) and fractures of the mandibular angle and condyle using panoramic radiographs to offer valuable data references for oral clinical research.

**Material and Methods:**

A retrospective cross-sectional study was undertaken, involving the collection of 409 cases of mandibular fracture in the Yanbian University Hospital. The case records and panoramic radiographs of mandibular angle fracture (78 cases) and condylar fracture (106 cases) were evaluated.

**Results:**

In the comparative analysis between the mandibular angle fracture group and the condylar fracture group, statistical significance was observed in the variables of M3 existence (*P* = 0.002), eruption of M3 from the alveolar cavity (*P* = 0.003), P&G position classification (*P* = 0.001), deep impactions (Classes IC, IIC, IIIB, and IIIC) (*P* < 0.001), and the presence of impacted M3 in both groups (*P* < 0.001).Regarding M3 roots, the mandibular angle fracture group exhibited the highest prevalence of multiple roots at 75.4%, surpassing the 64.6% observed in the condylar fracture group. The prevalence of proximal angles in the mandibular angle group and the condyle group was the highest, accounting for 64.6% and 61.5%, respectively. The percentage of M3 in the two groups was 80% and 43.1%, respectively, with a significant difference (*P* < 0.001).

**Conclusions:**

Impacted mandibular third molars (M3) elevate the risk of mandibular angle fractures, while their absence or normal eruption reduces this risk and protects against condylar process fractures. The fracture risk is influenced by the M3's position: P&G Class II and Class B impactions, where M3s emerge partially from the alveolar bone, are significantly associated with mandibular angle fractures. In contrast, the absence of M3 or its placement in P&G Class I and Class A positions tends to correlate with a higher incidence of condylar process fractures.

** Key words:**Condylar fracture, mandibular angle fracture, panoramic radiographs, risk of fracture, third molar.

## Introduction

The human mandible is crucial for facial aesthetics, speech, chewing, and swallowing (1,2). It's the most robust among facial bones but more prone to fractures, comprising 19%-40% of all facial fractures, second only to the nose (3). High fracture rates, especially in the condylar process (56.5%-63.2%) and mandible angle, are attributed to the bone's weak areas, notably the thin condylar process neck. Factors affecting fracture risk include external force direction and magnitude, biomechanical traits like bone density and pathological weakening, masticatory muscle force, and occlusal pressure (1,2,4-6). Predominantly, males (84%-86%), aged 28-30, face such fractures, mainly due to traffic incidents and violence (3,7-9).

Research has revealed that 88.9% of individuals with mandibular angle fractures possess impacted third molars (M3), while 59.5% of those with condylar process fractures do not have impacted M3 (5). These findings have piqued the interest of maxillofacial surgeons, prompting preliminary confirmation from researchers about the correlation between M3 and fractures of the mandibular angle and condylar process. Despite these insights, the medical community has yet to reach an agreement on how the position of impacted M3 influences these types of fractures (10,11). This study compiled data from 409 instances of mandibular fractures, which include 78 mandibular angle fractures and 106 condylar process fractures. It meticulously recorded each patient's age, gender, the cause of the fracture, and radiographic images. The investigation aims to discern the relationship between the position of the third molar (M3) and mandibular fractures, specifically at the angle and condylar process. It further examines how the presence, eruption status, and position of M3 affect these fractures, with the objective of offering a data reference to inform and enhance maxillofacial surgical practices.

## Material and Methods

- Research participants

The study involved the collection of hospitalized cases and panoramic radiographs of patients diagnosed with mandibular fracture at Yanbian University Hospital between 2012 and 2023. Data pertaining to the sex of the patient, age, cause of injury and location of mandibular fracture were recorded. The collected data was statistically analyzed using SPSS26.0 software. There were 409 cases in the study cohort, including 78 cases diagnosed with mandibular angle fracture and 106 cases diagnosed with condylar fracture. The correlation between M3, and mandibular angle and condylar fracture was analyzed by using panoramic radiographs.

Exclusion criteria: Edentulous jaw, incomplete medical records, and underdiagnosed cases.

- Standards for analyzing the content

The eruption status of M3 is defined as follows:

Eruption refers to the highest point on the mandibular third molar (M3) that passes through alveolar bone.

Non-eruption refers to the highest point on the M3 that did not penetrate through the alveolar bone.

Based on the outcomes derived from clinical and panoramic radiographs, the vertical position (Class A, Class B, Class C) and horizontal position (Class I,Class II,Class III) of the M3 were categorized according to the Pell and Gregory classification (P&G).

In addition, the impacted molars were divided into two groups (12):

Superficial impaction cases (class IA, IB, IIA, IIB, or IIIA);Deep impaction cases (class IC, IIC, IIIB, or IIIC)

Definition of the angle of M3: The angle between the longitudinal axis of the tooth and the occlusal plane.

**Figure 1:**
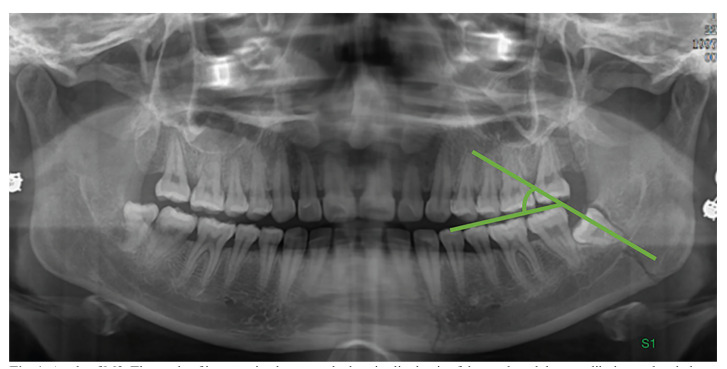
Angle of M3: The angle of intersection between the longitudinal axis of the tooth and the mandibular occlusal plane.

According to Ma'aita and Alwrikat (13), the classification is as follows (Fig. 1): Distoangular: more than100°; Vertical: 81 to 100°; Mesioangular: 21 to 80°; Horizontal: less than 20°

**Figure 2:**
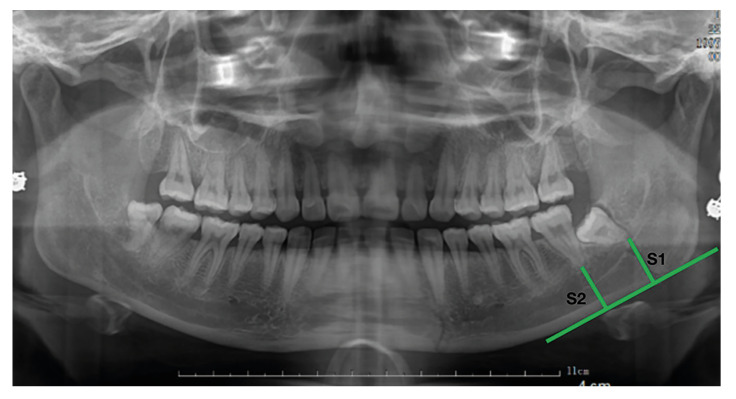
The position of M3 relative to the inferior border of the mandible.

-Position of M3 relative to the mandibular margin (Fig. 2),

By comparing the shortest distance between M3 and the inferior border of the mandible and the adjacent second molar, it can be divided into two categories:

Class 1: The shortest distance (S1) between M3 and the inferior border is equal to or longer than the second molar (S2); Class 2: The shortest distance between M3 and the inferior border is shorter than that of the second molar.

**Figure 3:**
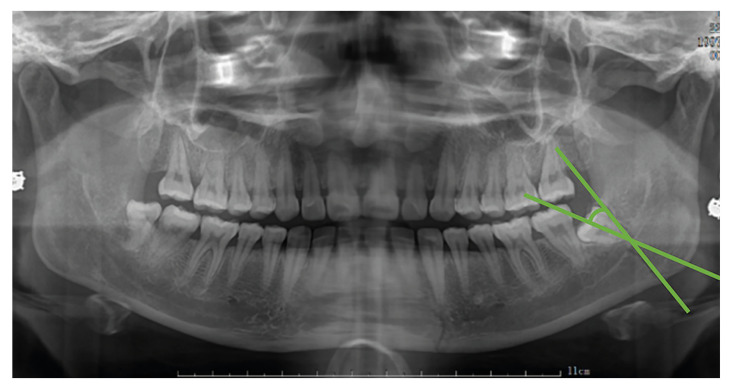
The angle between the fracture line of the angle of mandible and the longitudinal axis of the M3.

Angle between the line of fracture of the angle of mandible and the longitudinal axis of the M3 (Fig. 3)

The angle between the extension line opposite the fracture line of the angle of mandible and the longitudinal axis of the third molar (M3).

Determination of the number of visible roots on the x-ray film as follows:

Edentulous root formation; Single (conical root);Two or more roots.

M3 is impacted or not:

Impacted eruption;Normal eruption.

Analysis content:

Sex, age, time of onset and cause of injury of mandibular angle fracture and condylar fracture.The relationship between the position of mandibular M3 and mandibular angle fracture and condylar fracture.All the data are inputted into the computer and analyzed by SPSS26.0 software.

## Results

Statistically significant differences were observed between the two groups in various factors, including the presence or absence of mandibular M3 (*P* = 0.002), whether M3 emerged from alveolar bone (*P* = 0.003), P&G position classification (*P* = 0.001), deep impactions (Classes IC, IIC, IIIB, and IIIC) (*P* < 0.001), and whether M3 is impacted (*P* < 0.001). There were no statistical significances between the angle between M3 and the combined plane, and the type of fracture.

- Statistical results of the sample general data (Table 1)

The sample population consisted of 409 individuals diagnosed with mandibular fracture, with the male to female ratio of 3.65:1, and the average age was 37.46 ±15.56 years. The injuries were more frequent in summer and autumn. Traffic accident injuries were the highest (accounting for 37.65% of mandibular fracture, male to female ratio of 3.16:1), followed by falls (accounting for 32.27% of mandibular fractures, male to female ratio of 2.77:1), violence-related injuries (male to female ratio of 7.29:1). Additionally fall-related injuries and smashing injuries were also observed as causes of mandibular fractures.

In the group diagnosed with mandibular angle fracture, there were 78 individuals, with an average age of 32 years, and the ratio of male to female was 4.2:1. The occurrence of injuries was frequent in autumn season. The traffic accident-related injuries were the highest (38.46%), followed by violence-related injuries (30.77%). The ratio of left and right sided mandibular angle fractures was 3.11:1, and the left side fractures accounted for 75.64% of the total mandibular angle fractures. M3 was observed to be along the fracture line, accounting for 83% of the total number of mandibular angle fractures. This difference was statistically significant in comparison with condylar fracture (*P* = 0.002).

There were 106 individuals in the condylar fracture group, with an average age of 32, and the ratio of male to female was 2.4: 1. The injuries occurred frequently in summer season. Fall-related injuries being the highest (66.98%), and followed by traffic accident-related injuries (19.81%). In 62% of the individuals, the presence of M3 was associated with the fractures.

- Comparative analytic results of mandibular angle fracture group and condylar fracture group in the sample population (Table 2, Table 3):

- M3 results of horizontal and vertical positions

In the mandibular angle fracture group, the prevalence of P&G II fractures were the most common (52.3%), followed by P&G I fractures (32.3%). Class B accounted for the most (49.2%), followed by Class A (32.3%). M3 alveolar bone accounted for 96.9%.

In the condylar fracture group, P&G I fractures accounted for the most (61.5%), followed by P&G II fractures (23.1%). Class A accounted for the most (61.5%), followed by Class B (21.5%). M3 alveolar bone accounted for 80%. The *P* values of horizontal position and vertical position of the two groups of samples were all equal to 0.001, which is statistically significant. Class II had an OR value of 0.232, 95% CI, with a range of 0.104-0.518). Class B had an OR value of 0.230, 95% CI, with a range of 0.101-0.522).

-The result of the angle of M3

The median angle between the occlusal plane of mandibular angle fracture and the long axis of tooth was 50° (with a range of 26.90-97.10). The median angle between the occlusal plane of condylar fracture and the long axis of tooth was 69° (with a range of 46.50-96.2).

-Results of the number of roots in M3

The prevalence of multiple roots in the mandibular angle fracture group was the highest at 75.4%, compared to 64.6% in the condylar fracture group (OR value of 0.529, 95% CI with a range of 0.158-1.161).

- Vertical distance from the lowest point of the roots of M3 to the inferior border of the mandible

The distance of S1 in the mandibular angle fracture group was 38.5%, and that in the condylar fracture group was 40%. The median value of M3 distance (S1)/M2 distance (S2) in the mandibular angle fracture group was 1.05 (with a range of 0.87-1.49), and the median of S1/S2 in condylar fracture group was 1.04 (with a range of 0.93-1.35).

- The results of the angle between the long axis of the tooth and the fracture line in the mandibular angle fracture group

The median angle between the long axis of teeth and fracture line in the group of patients with mandibular angle fracture was 16° (with a range of 8.50-66.70).

- The result of whether M3 is impacted

The incidence of impacted mandibular M3 in the mandibular angle fracture group is 80%, whereas in the condylar fracture group, it is 43.1%, indicating a statistically significant difference (*P* < 0.001; OR value of 0.189, 95% CI: 0.087-0.413). P&G Class II and Class B impacted states are significantly associated with mandibular angle fractures. In contrast, M3 deletion, P&G Class I, and Class A positions are significantly linked to condylar fractures.

## Discussion

Among the 409 patients diagnosed with mandibular fractures, with males constituting 78.5% of the total population. Injuries occurred most frequently during the summer and autumn seasons. Among these, traffic accident injuries, including those involving bicycles and motorcycles, were the most common, followed by fall injuries, violence-related injuries, injuries from high falls, crush injuries, sports-related injuries, and injuries caused by blunt instruments. Among mandibular fractures, condylar fractures emerged as the most prevalent, with mandibular angle fractures ranking second, aligning with the research findings reported by Hagan *et al*. 62 years ago (14) (Table 1).

The study encompassed a total of 78 participants diagnosed with mandibular angle fractures, representing 80.8% of the male population. The highest incidence of injuries occurred during the autumn season, followed by the summer season. The patients with mandibular angle fractures were mostly between 20 and 40 years old, with an average age of 32 years, ranging from 22.75 to 39.00 years. According to studies, patients with mandibular angle fractures are statistically younger than those without mandibular angle fractures, ranging from 29.5 to 30.8 years (15).The primary etiology of injury is attributed to traffic accidents, followed by violent injury and fall injury. Due to the increasing number of motor vehicles, the prevalence of mandibular angle fractures from traffic accidents is more than violent injuries, with a ratio of 1.25:1. Hence, certain studies have observed that low-intensity traumatic impacts are more prone to causing mandibular angle fractures, particularly in the presence of the M3 (16).The prevalence of left side mandibular angle fractures was found to be 75.6%, with an additional 30.8% of cases exhibiting combined fractures including the chin. Two cases were reported to have fractures of the body of the mandible, all of which were observed in female patients. Various studies have consistently indicated a noteworthy incidence of left-sided mandibular angle fractures. The observed odds ratio of violent injuries suggests a potential association with the perpetrator’s right-handedness (17).

The study had a total of 106 participants with condylar fracture. The males constituting 70.8% of the cases, with an average age of 32 years, ranging from 21.00 to 42.25 years. The leading cause of injury was falls, followed by traffic accidents, high falls, collisions, and sports-related injuries. A trend was observed where sports injuries were more prevalent among females, while aggressive and shattering injuries were more commonly noted among males. The incidence was higher during the summer season, followed by the autumn season. The distribution of condylar fractures by side revealed a ratio of 1.6:1.7:1 for left, right, and bilateral sides, with 43.4% of cases being complicated by associated chin fractures. Notably, all patients with complicated body fractures were women.

There was no statistical significance in the etiology and gender distribution of the two groups, which is consistent with the research of Brucoli (18). A study by Semel revealed that the main cause of mandibular angle fracture was forceful trauma, while the fractures in the condylar group were mainly a result of falls (19). The results of our study indicated that falls were the predominant cause of condylar fractures, aligning with the findings reported by Nogami. Additionally, Nogami proposed that a substantial force is necessary to induce a condylar fracture (20). However, it has been posited that mandibular M3 may lose their significance in contributing to mandibular angle fractures when the mandibular angle is subjected to a substantial force (21). During the early 1980s, some scholars, such as Reitzik, used monkeys in a study to investigate fractures of the angle of mandible. They discovered that the force causing mandibular angle fractures in the M3 group was only 60% of that observed in the non-M3 group (14). Combined with our research, we have reason to speculate that the lower force of impact force on the angle of mandible (such as violent injury) is a risk factor for mandibular angle fracture.

No noTable correlation was identified between the M3 and the occlusal plane angle or the type of fracture. The median angle between the occlusal plane and the long axis of the tooth in the mandibular angle fracture group was 50°, while in the condylar fracture group, it was 69°.The most prevalent angle range in both the mandibular angle group and the condylar process group was the close angle range (21°-80°), constituting 64.6% and 61.5%, respectively (Table 2).

Multiple mandibular angle fractures (defined as having a number of roots ≥ 2) constituted 75.4%, while condylar fractures accounted for 64.6%. Nevertheless, certain studies have reported a noteworthy association between a mandibular third molar with a single root and mandibular angle fractures (Table 2). The hypothesis posited is that stress concentration around a single root tip may exceed the strength of the surrounding bone (22).

In addition to the presence of the M3, its position is a contributing factor to the occurrence and location of mandibular fractures. The prevalence of the third molar is 83.3% in the mandibular angle fracture group and 62.3% in the condylar fracture group (*P* = 0.002). There are statistical differences between the two groups in the presence or absence of M3 (*P* = 0.002), the emergence of M3 from alveolar bone (*P* = 0.003), the classification of P&G position (*P* = 0.001), deep resistance (IC level, IIC level, IIIB level, and IIIC level) (*P* < 0.001) and the impaction of M3 (*P* < 0.001). The presence of an impacted M3 (P&G II and B) in the alveolar bone is significantly associated with the mandibular angle fracture, while the absence of M3, P&G I, and A position are significantly related with condylar fracture (Table 3, Table 4).

Consistent with the findings of this study, other research has identified a significant association between P&G Class II or III and Class B impactions with mandibular angle fractures. Conversely, the absence or full eruption (Class IA) of the third molar has been significantly linked to fractures of the condylar process (23). Meta-analyses by researchers have demonstrated that a Class B impacted M3 elevates the risk of mandibular angle fractures. When comparing Class B with Class A impactions, the more deeply positioned Class B M3 implies greater bone fragility in that area for affected patients. The text examines how M3, depending on their presence and classification, influence the likelihood of fractures in the mandibular angle. It notes an unexpected finding: Category C M3 do not elevate the risk of fractures in comparison to Categories A and B (24). Additionally, it highlights a meta-analysis by Armond *et al*., which indicates that having an M3 increases the risk of angle fractures. This analysis also pinpoints the positions of M3 that are most and least conducive to such fractures (5,24,25).The meta-analysis by Armond *et al*. reveals that having a third molar (M3) can increase the likelihood of angle fractures by 3.27 times. Specifically, Category B and Class II impactions of M3 are identified as the most predisposing positions for such fractures, while Category A and Class I are considered protective. This research marks the first time the relationship between M3 impaction type and the incidence of both mandibular angle and condylar fractures has been investigated. Notably, all cases involving simultaneous mandibular angle and condylar fractures featured vertically impacted M3s (Class I and Category A impactions), with M3 positioned more superficially in these patients (5).All instances of concurrent mandibular angle and condylar process fractures stem from severe mandibular fractures, indicating that significant traumatic force is likely the primary cause for simultaneous fractures in both regions (25). Thus, it is speculated that under the conditions of heavy impact forces, the presence of the third molar has a relatively minor influence on the occurrence of fractures.

A meta-analysis revealed a significant correlation between mandibular angle fracture and M3, especially in P&G C, II and III, where M3 did not erupt completely (26). It has also been reported that mandibular angle fractures mainly occur in Class B, Class C, Class II and Class III (12,22,27) . However, according to some studies, individuals without erupted alveolar bone in the M3 region were more prone to have mandibular angle fractures than those who have erupted alveolar bone (28), which is contrary to our research findings. Nevertheless, certain studies have revealed that mandibular angle fractures were more prevalent in patients with M3 that did not erupt from the alveolar bone compared to those who did experience eruption (29).In other studies, it has been noted that the loss of M3 is correlated to the increased risk of condylar fracture, and the existence of M3 increases the incidence of mandibular angle fracture by 2.7 times. The risk of mandibular angle fracture varies with the position of the third molar, especially when M3 is not completely erupted (18,22,27). According to Thangavelu, having an impacted third molar increases the possibility of condylar fracture and puts the patient at risk for mandibular angle fracture.However, the absence of impacted M3 increases the risk of condylar fracture (29).To summarize, the impacted M3 is a risk factor for mandibular angle fracture, while an absence and normal eruption of M3 is a protective factor for the condyle.

Some clinical research has established that impacted M3 elevate the risk of mandibular angle fractures while concurrently lowering the risk of condylar fractures (10,11). However, several studies indicate that many researchers have overlooked potential influencing factors, such as the direction of the traumatic force and the point of impact. Finite element analysis suggests that the presence of an M3 tooth marginally elevates stress in the mandibular angle region but reduces it in the condyle. Conversely, models lacking an M3 show decreased stress in the mandibular angle and increased stress in the condyle (25). Two studies evaluated the effects of the presence of M3 and the direction of applied forces, with results indicating that M3 acts as a protective factor in cases of frontal and contralateral forces, while it decreases resistance to ipsilateral forces (22,25). In other words, the vulnerability of the mandibular angle and condylar regions depends on the presence of unerupted M3s, as well as the direction and point of force application (25). Scholars have highlighted external factors like the impact's magnitude and direction, and the uncertainty surrounding the shape of the impacting object. Additionally, they note that *in vitro* studies fall short of replicating patient-specific factors such as dental alignment, the position of the mandibular bone, and the effects of related soft tissues. Thus, only clinical studies can fundamentally provide the basis for understanding the mechanisms behind fracture occurrence. Thus, only clinical studies can fundamentally provide the basis for understanding the mechanisms behind fracture occurrence (5).

**Figure 4:**
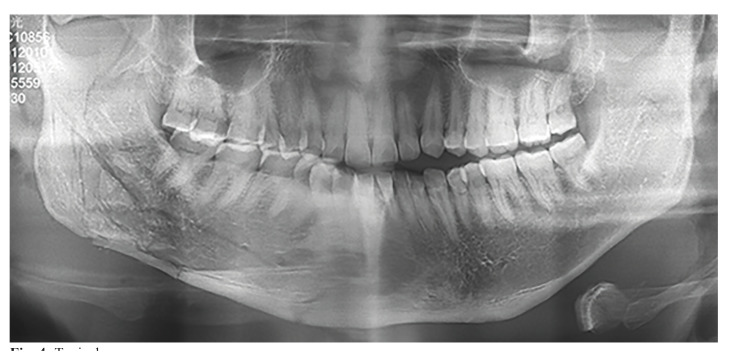
Typical case.

Some scholars have suggested the preemptive removal of M3, particularly for individuals involved in contact sports. However, it has been observed that similar cases exist where the mandibular angle bone was thick, and M3 erupted in the correct position (Fig. 4). When the mandibular angle is affected, a comminuted fracture rather than a linear fracture occurs, making the procedure more challenging. According to our findings, the absence of M3 eruption appears to provide protection to the condyle, whereas impacted M3 represents a risk factor for mandibular angle fractures. In our clinical cases, we hypothesize that this relationship may be linked to stress interruption. Another perspective is that mandibular angle fractures could potentially absorb stress on the condyle, thereby potentially reducing the incidence of condylar fractures. Furthermore, certain scholars have identified that the incidence of mandibular angle fractures tends to increase in individuals with a high mandibular angle (19,30). Therefore, the prophylactic extraction of the third molar without clinical symptoms warrants careful consideration. Clinicians should assess the patient's clinical symptoms comprehensively before making decisions on whether to retain or extract the third molar.

To summarize, the presence and location of the mandibular third molar (M3), particularly in P&G Class II and Class B, along with minor external forces, such as those from violence, act as risk factors for mandibular angle fractures and protective factors for the condylar process. While *in vitro* studies lay a groundwork for understanding fractures, they fall short of capturing individual patient factors such as dental condition, mandibular position, and the condition of associated soft tissues. Thus, it is recommended that research ultimately leans on experimental studies, integrated with clinical observations, to more effectively uncover the mechanisms behind maxillofacial fractures. The understanding of fracture mechanisms, including the presence and location of the third molar (M3), the force's direction, magnitude, and point of application, along with patient-specific factors like gender and bone quality, requires additional clinical data and further experimental research. This enhanced data collection and analysis will offer more precise support for clinical practices.

## Conclusions

1.Impacted mandibular third molars (M3) increase the risk of fractures in the mandibular angle, whereas the absence of M3 or its normal eruption serve as protective factors against fractures in the condylar process.

2.Additionally, smaller forces, like those resulting from violence, impacting the mandibular angle pose a risk factor for fractures in this area.

3. Left-side mandibular angle fractures occur more frequently, and this trend could be linked to the predominance of right-handedness among assailants, as suggested by the odds ratio for injuries resulting from violence.

4.The risk of fractures changes based on the position of the third molar (M3); impacted third molars emerging from the alveolar bone, specifically P&G Class II and Class B impactions, are strongly linked to fractures of the mandibular angle. Conversely, the absence of M3, along with P&G Class I and Class A positions, are closely associated with fractures of the condylar process.

## Figures and Tables

**Table 1:** General information on mandibular fractures.

Facture etiology	Detection rate (%)			Age		
	Male (n=321)	Female (n=88)	*P*	Male (n=38)	Female(n=35.5)	*P*
Traffic accident	117 (36.4)	37 (42.0)	0.106	39.5 (29.0-49.0)	41.0 (24.5-51.0)	0.930
Assault	51 (15.9)	7 (8.0)	-	36.0 (23.0-45.0)	25.0 (19.0-34.0)	0.123
Fall	97 (30.2)	35 (39.8)	-	35.5 (26.0-51.0)	28.0 (21.0-44.0)	0.080
High fall	17 (5.3)	6 (6.8)	-	37.0 (28.5-44.5)	43.0 (10.5-53.8)	0.806

^a^p-Value is 0.0001 (p < 0.05) and is statistically significant.

**Table 2:** The measurement results of mandibular angle fractures and condylar process fractures.

Group		P25	P50	P75	P90	P95
angle fracture group	Angle between M3 long axis and fracture line	8.50	16.00	33.30	51.04	67.70
	S1/S2	0.87	1.05	1.24	1.37	1.49
	Angle of occlusal plane and M3 longitudinal axis	26.90	50.00	74.10	92.00	97.10
condylar fracture group	S1/2M	0.93	1.04	1.16	1.28	1.35
	Angle of occlusal plane and M3 longitudinal axis	46.50	69.00	81.00	89.40	96.20

P50 represents the median ;Percentile (P).

**Table 3:** Comparative values of the mandibular angle fracture and condylar fracture groups.

Variables		Angle Fracture Group	Condylar Fracture Group	*P*	OR (95% confidence interval)
Gender, n (%)	Male	63 (80.8)	75 (70.8)	0.121	1.736(0.861-3.501)
	Female	15 (19.2)	31 (29.2)		
Age, yr, n (%)	Range	32.00 (22.75-39.00)	32.0 (21.00-42.25)	0.895	1.007(0.985-1.029)
Existing M3, n (%)	Yes	65 (83.3)	66 (62.3)	0.002	0.330(0.162-0.673)
M3 erupted from alveolar bone, n (%)	Yes	63 (96.9)	52 (80.0)	0.003	0.127(0.027-0.588)
Horizontal,n (%)	I	21 (32.3)	40 (61.5)	0.001	1
	II	34 (52.3)	15 (23.1)		0.232(0.104-0.518)
	III	10 (15.4)	10 (15.4)		0.525(0.189-1.461)
Vertical, n (%)	A	21 (32.3)	40 (61.5)	0.001	1
	B	32 (49.2)	14 (21.5)		0.230(0.101-0.522)
	C	12 (18.5)	11 (16.9)		0.481(0.182-1.274)
Number of roots, n (%)	Absent	7 (10.8)	14 (21.5)	0.238	1
	Single root	9 (13.8)	9 (13.8)		0.500(0.137-1.825)
	Follow more	49 (75.4)	42 (64.6)		0.529(0.158-1.161)
S1/S2, n (%)	Class 2	25 (38.5)	26 (40.0)	0.857	0.938(0.464-1.896)
Angle of occlusal plane and M3 longitudinal axis, (%)	Distoangular (>100°)	1 (1.5)	2 (3.1)	0.229	1
	Vertical(81-100°)	11 (16.9)	18 (27.7)		0.818(0.066-10.117)
	Mesioangular (21-80°)	42 (64.6)	40 (61.5)		0.476(0.042-5.459)
	Horizontal (≤20°)	11 (16.9)	5 (7.7)		0.227(0.016-3.131)
Impacted, n (%)	Yes	52 (80.0)	37 (43.1)	<0.001	0.189(0.087-0.413)

^a ^*p-Value* is 0.0001 (*p* < 0.05) and is statistically significant.

**Table 4:** Correlation between mandibular third molar position and the mandibular angle and condylar fractures.

Position of third molar	Angle fracture	%	Condyle fracture	%	Total	%
Class I position A	17	30.4	39	69.6	56	43
Class I position B	4	80	1	20	5	3.8
Class I position C	1	100	0	0	1	0.8
Class II position A	0	0	1	100	1	0.8
Class II position B	26	65	14	35	40	30.8
Class II position C	5	100	0	0	5	3.8
Class III positionA	4	100	0	0	4	3.1
Class III position B	3	75	1	25	4	3.1
Class III position B	3	75	1	25	4	3.1
Class III positionC	4	28.6	10	71.4	14	10.8
Total	64	49.2	66	50.8	130	100

χ2 =28.590^ a^, *P*<0.0001; ^a ^*p-Value* is 0.0001 (*p* < 0.05) and is statistically significant.

## Data Availability

The datasets used and/or analysed during the current study available from the Correspondence on reasonable request. We declared that materials described in the manuscript, including all relevant raw data, will be freely available to any scientist wishing to use them for non-commercial purposes, without breaching participant confidentiality.
